# Patient experience and healthcare utilization for a COVID-19 telemedicine home monitoring program offered in English and Spanish

**DOI:** 10.1371/journal.pone.0270754

**Published:** 2022-06-30

**Authors:** Keri B. Vartanian, Megan Holtorf, Emily J. Cox, George Diaz, Hargobind Khurana, Sherene Schlegel, Caroline Raganit, Brandon Ong, Todd Czartoski

**Affiliations:** 1 Center for Outcomes Research and Education, Providence, Portland, OR, United States of America; 2 Providence Medical Research Center, Providence Health Care, Spokane, WA, United States of America; 3 Section of Infectious Diseases, Providence Regional Medical Center Everett, Everett, WA, United States of America; 4 Providence Research Network, Renton, WA United States of America; 5 Elson S. Floyd College of Medicine, Washington State University, Spokane, WA, United States of America; 6 Providence Telehealth, Renton, WA, United States of America; University of Oklahoma Health Sciences Center, UNITED STATES

## Abstract

**Background:**

Telemedicine is a vital component of the healthcare system’s response to COVID-19. In March of 2020, Providence health system rapidly implemented a telemedicine home monitoring program (HMP) for COVID-19 patients that included use of at-home pulse oximeters and thermometers and text-based surveys to monitor symptoms. By June 2020, Providence updated the HMP to be offered in Spanish. This program was implemented before COVID-19 testing was readily available and therefore was offered to all patients suspected of having COVID-19. This study examines engagement, experience, and utilization patterns for English and Spanish-speaking patients engaged in the COVID-19 HMP.

**Methods:**

A retrospective review of program data was used to understand HMP patient engagement (responsiveness to three daily text to monitor symptoms), satisfaction with the program (likelihood to recommend the program) as well as comfort using home monitoring devices and comfort recovering from home. To understand impact on care for COVID-19 confirmed cases, we used electronic health records to measure patterns in healthcare use for COVID-19 positive HMP participants and non-HMP propensity weighted controls. All patients enrolled in the COVID-19 HMP from March–October 2020 were included in the study. Patients tested for COVID-19 during the time window and not enrolled in HMP were included in the propensity-weighted comparison group. Descriptive and regression analyses were performed overall and stratified by English and Spanish speakers.

**Results:**

Of the 4,358 HMP participants, 75.5% identified as English speakers and 18.2% identified as Spanish speakers. There was high level of responsiveness to three daily text-based surveys monitoring symptoms engagement (>80%) and a high level of comfort using the home monitoring devices (thermometers and pulse oximeters) for English- and Spanish-speaking participants (97.3% and 99.6%, respectively). The majority of English (95.7%) and Spanish-speaking (100%) patients felt safe monitoring their condition from home and had high satisfaction with the HMP (76.5% and 83.6%, respectively). English and Spanish-speaking COVID-19 positive HMP participants had more outpatient and emergency departments (ED) encounters than non-participants 7 and 30 days after their positive test.

**Conclusion:**

This widely implemented HMP provided participants with a sense of safety and satisfaction and its use was associated with more outpatient care and ED encounters. These outcomes were comparable across English and Spanish-speakers, highlighting the importance and potential impact of language-concordant telemedicine.

## Introduction

COVID-19 infection can have severe symptoms, with potentially fatal consequences, that require intensive inpatient monitoring and care [1]. However, most COVID-19 cases are low acuity and do not require hospital admission [2, 3]. Keeping low acuity COVID-19 patients in the hospital longer than necessary to monitor symptoms is often unnecessary and ends up filling valuable hospital beds, consuming staff time, depleting personal protective equipment, and increasing risk of spreading the infection [4]. Thus, most often newly diagnosed low acuity COVID-19 patients need to monitor their condition from home as symptoms either resolve or worsen.

In response to this need for monitoring COVID-19 patients at home, several health systems rapidly developed telemedicine programs that provide home monitoring for COVID-19 patients. This included standing up protocols for patient assessment and referrals, deployment of home monitoring devices such as pulse oximeters, creation of electronic-based patient questionnaires to monitor symptoms, training of healthcare workers to engage in COVID-19 telemedicine care, preparing tracking systems to understand changes in patient symptoms, and rapid referrals to any needed in-person care. Health systems have deployed telemedicine home monitoring programs (HMPs) for patients exiting emergency department, in-patient care, and clinical settings [5–11]. Early investigation into these HMPs have provided key lessons on implementation, patient engagement, referrals to additional care, and even impacts on outcomes such as reduction in short-stay hospital admissions [5–11]. This research has begun to shape our understanding of COVID-19 home monitoring; however, many studies so far have key limitations such as having a small sample size, limited investigation into the patient experience with COVID-19 telemedicine HMPs, and lack of appropriate comparison groups.

Additionally, a key challenge with telemedicine is ensuring equitable practice and implementation as there is a digital divide in access and comfort with technology as well as language and cultural barriers to care [12]. For example, within an academic health system, not being an English speaker was associated with lower odds of completing telemedicine visits [13]. This is particularly concerning for the implementation of COVID-19 telemedicine HMPs as extensive data demonstrates the disproportionate impact of COVID-19 infection on people of color and those whose primary preferred language is not English [14–18]. Studies have shown twice the odds of testing positive for COVID-19 for Hispanic/Latino patients and among individuals whose primary preferred language is not English [15, 16, 18]. Thus, to overcome barriers to care, it is essential that telemedicine is offered in multiple languages and meets the needs of these populations. To our knowledge, no current studies of COVID-19 telemedicine HMPs have included services in multiple languages or consideration of outcomes by primary preferred language.

In March 2020, the Providence system telemedicine team promptly developed a HMP for COVID-19 patients [19]. The English language HMP was initially launched in March in Washington state and by May, was live in five states. Briefly, patients were enrolled in the program from the ED, inpatient units, urgent care clinics and COVID-19 clinics if they tested positive or were under suspicion for COVID-19, and a provider assessed the patient and determined that home monitoring was appropriate versus hospital admission. Prior to going home, patients were consented for enrollment in the HMP, received education based on their primary language, and were given an FDA-approved digital battery-operated pulse oximeter (Finger Soft Digital Pulse Oximeter, Medline) and thermometer (Large Display Digital Thermometer, Medline) to self-report vital signs (oxygen saturation, respiratory rate, heart rate, temperature) and symptoms. Data were initially reported three times a day for two weeks via a SMS text-based patient questionnaire (via Twistle, a HIPAA compliant patient engagement platform operated by Health Catalyst). Vital sign thresholds were rated on a point system contributing to an overall severity alert score to visually assist clinicians in prioritizing concerning vital signs on a clinical dashboard. Four severity levels were created: green within normal limits, yellow at high normal or at low normal limits, red above highest normal or below lowest normal, and red flag superseding all scores when a patient self-reported oxygen saturation of 88% or less requiring immediate response from the clinical team. Nurses reviewed a dashboard which depicted vital signs as red, yellow or green based on severity of reported data. Patients reporting concerning vital signs (scored as red or yellow) received a follow up phone call and or a video call from a home monitoring telemedicine nurse to assess and triage. Interpreter services were an integral component of patient engagement and included ensuring live interpreters were available 24/7 for the emergency department (ED) or the command center nurses to connect with the patient in 200+ languages. The Spanish language HMP was launched in June of 2020, which specifically provided SMS text-based questionnaires in Spanish.

The purpose of this project was to understand engagement and patient satisfaction with the Providence COVID-19 HMP among English and Spanish speakers. Additionally, we evaluated use of healthcare for the HMP COVID-19 positive patients in comparison to similar COVID-19 positive patients not engaged in HMP, overall and stratified by English and Spanish speakers to understand connection to care.

## Methods

### Ethics approval

This project comprised a retrospective review of a telemedicine HMP intervention that was implemented in 2020 across the Providence system. The retrospective analysis was approved by the Providence Institutional Review Board, and the requirement for informed consent was waived.

### Retrospective data collection

For this retrospective cohort study, we used electronic health records from the Providence St Joseph health system which includes a mix of hospitals, urgent care facilities, and outpatient clinics. Available data include demographics, diagnosis, medications, procedures, lab results, and vital signs. Electronic health records were accessed for the study population to obtain final COVID-19 status, patient demographics, geographic region, COVID-19 encounter setting, and primary symptoms at the time of their visits when COVID-19 testing occurred. Subsequent primary care and ED use 7 and 30 days following their COVID-19 test was also obtained through electronic health records.

Data on symptoms, HMP experience, and satisfaction were collected through Twistle using SMS-based questionnaires. Self-reported symptoms were collected 2–3 times per day for 14 days and experience and satisfaction were collected on day seven post enrollment. Data collected through Twistle were then matched to electronic health records.

### Study population

**Fig 1:**
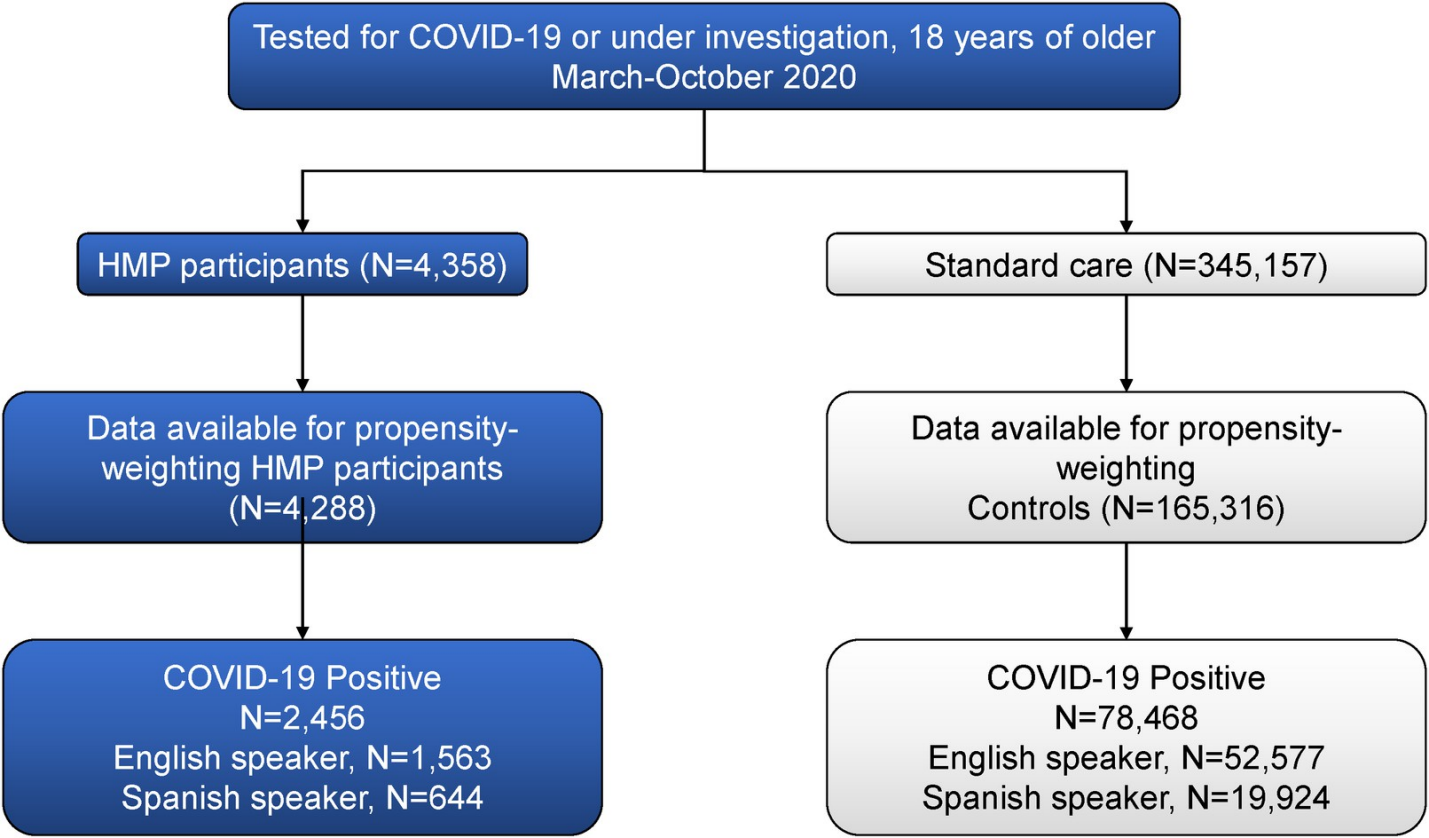
STROBE diagram. The flow diagram illustrates the retrospective selection of the COVID-19 home monitoring program (HMP) cohort and the control cohort. Propensity weighting was performed to minimize differences, so our sample was limited to individuals with the data elements needed for the weighting (see S1 Data for complete unweighted and weighted sample characteristics). Coefficients of the covariates were used to weight the regression model used to calculate risk of outpatient and primary care utilization and emergency department use. Final examination of COVID-19 positivity came from electronic health records as did patient primary preferred language. Of note, there were multiple primary languages in sample, which is why the breakdown of English and Spanish speaking patients do not sum to the total N for the HMP and standard care groups.

All Providence patients who received a COVID-19 test, or who were under investigation for COVID-19 between March 2020 and October 2020 and were 18 years of age or older at the time of their first COVID-19 related encounter were included in the study. Patients were assigned to cohorts based on either being a HMP participant (treatment) or a patient under standard care (control). We applied propensity weighting to the control group to account for differences between groups (see statistics section below for detail). Based on those with available data for propensity weighting, our final sample was 4,288 treatment and 165,316 control. Of those, 2,456 treatment and 78,468 control had a positive COVID-19 test **(Fig 1)**. Among the COVID-19 positive patients in the treatment group, 1,563 were English speakers and 644 were Spanish speakers. Among the COVID-19 positive control group, 52,577 were English speakers and 19,924 were Spanish speakers. There were other languages spoken among each group, which is why the English and Spanish speaking populations do not sum to the total N for each group.

### Outcomes

Engagement with the HMP was measured as the proportion of completed unique responses to the three daily text prompts for the enrolled HMP population within the first 14 days. Satisfaction and experience with the HMP were measured with a three-item SMS-based survey sent on day seven that included measures of whether the home monitoring devices (the pulse oximeter and thermometer) were easy to use, whether the home monitoring helped them feel safe recovering from home, and whether they would recommend the HMP to someone with COVID-19. Satisfaction was calculated as the proportion of respondents who would recommend the program (Score > = 9). Net Promoter Score was calculated using the proportion of promoters (score ≥ 9) minus the proportion of detractors (score ≤ 6). NPS scores range from -100 to100 with the following categories: -100-0 = needs improvement, 0–30 = good, 30–70 = great, and 70–100 = excellent [20].

For both the HMP and standard care group, healthcare utilization was assessed as the presence or absence of any encounter in electronic health records after the initial COVID-19 encounter. We calculated percent of patients with an outpatient ambulatory care visit, primary care physician (PCP) visit, or ED visit 7 and 30 days after date of COVID-19 test. PCP visits were defined as a subset of outpatient ambulatory care visits (both virtual and face-to-face) which occurred in a department that offers primary care services.

### Statistics

All analyses were performed using SAS software, version 9.0. For all analyses, P ≤.05 (two-tailed) were used as criterion for statistical significance. Descriptive statistics were used to summarize demographic and clinical characteristics of program participants as well as engagement and satisfaction with the HMP.

Propensity score weighting was used to minimize differences between HMP participants (treatment) and non-participants (controls) [21].To perform the score weighting, a logistic regression model was used to estimate the likelihood of participation in the HMP. The variables used in the logistic model included participant demographics, initial encounter location and department, past medical and utilization history, and presenting COVID-19 symptoms (see S1 Data for overview of variables included). The probability of participation in the HMP was defined using the results of the logistic regression. This probability was then used to construct a weight for subsequent analysis to adjust for differences between HMP participants and non-participants [21].

To determine the average treatment effects for the treated, probability weights were calculated where the weight is 1 for HMP participants and probability/(1-probability) for non-participants [22]. Weights were then normalized to equal the sample size in each group [22, 23]. The complete un-weighted and weighted variables for the HMP and non-HMP groups are available in the S1 Data.

To examine associations between program participation and any subsequent healthcare utilization, relative risks (RRs) and 95% confidence intervals (CI) were estimated using multivariable modified Poisson regression with robust standard errors [24]. Results were adjusted for age (continuous), race, Pneumonia Severity Index score (PSI, continuous), and smoking status. PSI was used as it has been shown to be predictive of COVID-19 severity [25]; however, we used a slightly modified version that excluded imaging, mental health exam, and hospice/long-term care as this was not present in the examination of majority of the COVID-19 patients. Propensity weights were applied to all analysis.

## Results

### Participants

A total of 4,358 patients participated in the HMP **(Table 1)**. English was spoken by 75.5% of HMP participants and Spanish was spoken by 18.2%. Among the HMP participants, 51.7% identified as female, 45.9% identified as White, 33.7% identified as Hispanic/Latino, 4.9% identified as Black, and 4.7% identified as Asian. A little less than half had their qualifying COVID-19 encounter in the emergency department (47.5%), while 31.8% came from inpatient care, and 17.2% came from outpatient care. Thirty percent had commercial insurance coverage, 27.2% were on Medicare, and 25% were on Medicaid. The most common symptom at presentation was shortness of breath (38.2%) followed by cough (34.5%). About half of the HMP participants tested positive for COVID-19 (56.4%).

**Table 1:** Home monitoring program participant characteristics.

	HMP (n = 4,358)		HMP (n = 4,358)
**Age**		**Encounter Setting**	
18 to 34	691 (15.9%)	ED	2,069 (47.5%)
35 to 44	672 (15.4%)	IP	1,384 (31.8%)
45 to 54	843 (19.3%)	OP	749 (17.2%)
55 to 64	878 (20.1%)	Virtual	156 (3.6%)
65 to 74	737 (16.9%)	**Symptoms at Presentation**	
75+	537 (12.3%)	Chills	2.9%
**Sex**		Congestion	1.6%
Female	2,254 (51.7%)	Cough	34.5%
Male	2,104 (48.3%)	Fever	26%
Other	0 (0.0%)	Flu-like symptoms	2.2%
Unknown	0 (0.0%)	GI symptoms	7.5%
**Race**		Headache	4.7%
American Indian or Alaska Native	41 (0.9%)	Hemoptysis	0.3%
Asian	207 (4.7%)	Infection	1.6%
Black	214 (4.9%)	Myalgia	6%
Hispanic or Latino	1,467 (33.7%)	Reactive Airway	0.1%
Native Hawaiian or Other Pacific Islander	100 (2.3%)	Shortness of breath	38.2%
Other	213 (4.9%)	Throat conditions	3.9%
Unknown	111 (2.5%)	Wheezing	0.2%
White	2,001 (45.9%)	**Final COVID status**	
**Language**		Negative	1,795 (41.2%)
English	3,292 (75.5%)	Positive	2,456 (56.4%)
Other	269 (6.2%)	PUI	0 (0.0%)
Spanish	793 (18.2%)	Unknown	107 (2.5%)
Unknown	4 (0.1%)		
**Payor Type**			
Capitation	164 (3.8%)		
Commercial	1,309 (30.0%)		
Managed Care	122 (2.8%)		
Medicaid	1,108 (25.4%)		
Medicare	1,185 (27.2%)		
Other	355 (8.1%)		
Self-pay	115 (2.6%)		

ED = emergency department; HMP = home monitoring program; IP = inpatient; OP = outpatient; PUI = Patient Under Investigation.

### Patient engagement and satisfaction with HMP

Engagement with the interface, defined as the average number of completed responses to the three times daily text prompts, was high (87.2 ± 23.3%) and was similar between Spanish- and English-speaking participants (77.6 ± 22.4 vs. 80.1 ± 20.9%) (**Table 2**). The vast majority of respondents indicated that devices were easy to use (97.7%, **Table 3**), and they felt safe recovering at home with the HMP (96.9%). The high percentage reporting ease of use and feeling safe recovering was true for English and Spanish-speakers. Overall, 79.1% of participants indicated 9–10 on the likelihood that they would recommend the HMP and the overall net promoter score was 71.5. Again, this high level of satisfaction was observed among both English and Spanish speakers (NPS = 67.4 and 77.5, respectively).

**Table 2:** Engagement.

	Mean	Std
**Overall**		
	87.2%	23.3%
**Language**		
English	80.1%	20.9%
Spanish	77.6%	22.4%

Engagement = as the proportion of complete unique responses across the HMP population to three daily text prompts within the first 14 days following program referral. Overall N = 4,358; English N = 3,292, Spanish N = 793.

**Table 3:** Home monitoring program participant satisfaction.

	Overall	English	Spanish
**Were the devices, pulse oximeter and thermometer, easy to use?**			
No	68 (2.3%)	33 (2.7%)	1 (0.4%)
Yes	2,834 (97.7%)	1,186 (97.3%)	248 (99.6%)
**With home monitoring, do you feel safe recovering at home?**			
No	89 (3.1%)	52 (4.3%)	0 (0%)
Yes	2,813 (96.9%)	1,167 (95.7%)	249 (100%)
**Would you recommend this COVID-19 home monitoring**			
Recommend (9–10)	2,295 (79.1%)	822 (76.5%)	208 (83.6%)
NPS	71.5	67.4	77.5

Results of a satisfaction survey sent to participants in the HMP. The third item was rated on a scale of 1 (Very Unlikely) to 10 (Very Likely). NPS = net promoter score.

### Care utilization

Overall, COVID-19 positive participants in the HMP were more likely to have an outpatient encounter compared to non-participants. For Spanish-speaking COVID-19 positive participants, the likelihood of both primary care and outpatient encounters was higher at 30 days compared to COVID-19 positive Spanish-speaking non-participants. For COVID-19 positive English-speaking participants, the likelihood of outpatient encounters was higher at both 7 days and 30 days, and the likelihood of primary care encounters were higher at 30 days compared to COVID-19 positive English-speaking non-participants **(Table 4)**. COVID-19 positive participants in HMP also were more likely to have an ED visit in the first 7 and 30 days following their COVID-19 diagnoses, and this was also true among COVID-19 positive English and Spanish-speaking participants.

**Table 4:** Care utilization after the initial COVID-19 encounter in COVID-19 positive home monitoring participants versus propensity-weighted COVID-19 positive controls.

	Overall		English		Spanish	
	RR (95% CI)	p-value	RR (95% CI)	p-value	RR (95% CI)	p-value
**Outpatient**						
**7 Days**	1.81 (1.31, 2.49)	0.000	2.03 (1.41, 2.91)	< .001	1.58 (0.80, 3.15)	0.190
**30 Day**	1.27 (1.09, 1.49)	0.003	1.28 (1.07, 1.54)	0.008	1.46 (1.02, 2.09)	0.038
**Primary Care**						
**7 Days**	1.09 (0.90, 1.30)	0.382	1.15 (0.93, 1.43)	0.193	1.20 (0.79, 1.83)	0.388
**30 Day**	1.42 (1.25, 1.62)	< .001	1.47 (1.27, 1.71)	< .001	1.61 (1.21, 2.16)	0.001
**Emergency Care**						
**7 Days**	2.07 (1.73, 2.49)	<0.001	2.22 (1.78, 2.76)	<0.001	2.11 (1.47, 3.03)	<0.001
**30 Days**	1.52 (1.32, 1.76)	<0.001	1.54 (1.30,1.83)	<0.001	1.72 (1.28,2.32)	<0.001

Adjusted relative risk calculated using generalized linear model with log link and Poisson distribution with robust estimates with 95% CI controlling for age (continuous), race, Pneumonia Severity Index score (continuous), and smoking status.

## Discussion

In this study, we describe a HMP for COVID-19 patients that was launched by the Providence health system in March 2020. This program included referrals from acute and ambulatory settings. Patients were trained on how to use pulse oximeter and thermometers to monitor vital signs at home and responded to a text-based patient questionnaire that assessed symptoms multiple times a day. While the HMP offered interpretive services in more than 200 language and multiple written languages, the text-based questionnaires were offered in English and Spanish. Both Spanish- and English-speaking patients reported high levels of engagement and satisfaction with the HMP including feeling safe monitoring their conditions from home and comfort with using the at home monitoring devices. The net promoter score was 71.5 overall (67.4 for English Speakers and 77.5 for Spanish speakers). Compared with their propensity weighted COVID-19 positive counterparts who received standard care, COVID-19 positive participants in the HMP had higher rates of outpatient ambulatory care and ED visits 7 and 30 days after their initial COVID-19 encounter.

This HMP featured the use of two home monitoring devices to measure symptoms–a pulse oximetry and thermometer. There has been some debate about the use of at home monitoring of pulse oximetry for COVID-19 patients. There were early concerns that measurement could be difficult for patients to implement and accuracy of the readings may be questionable especially as saturation levels fall below 90% [26]. However, technological advancements that have made these devices simple to use and new data validating at home measurement has made at home pulse oximetry more accepted as standard practice for safely monitoring blood oxygen levels [27–32]. In this study, we demonstrate that 97.7% of HMP patients reported that the pulse oximeter and thermometers were easy to use, which provides a critically important patient perspective. This high level of comfort was seen in both English and Spanish-speaking patients (97.3% and 99.6%, respectively). It is important to note that in-person training, web-based video links, and written materials on how to use these devices were provided to all participants and was offered in multiple languages. This may have helped to improve ease of use of the home monitoring devices and could be considered an important part of engaging participants in home monitoring.

NPS is a standard measure used in customer experience programs. Health systems have started to use NPS with patients as well to assess satisfaction with their experience, including with telemedicine [33–35]. The overall NPS for the HMP was 71.5, which is considered “excellent.” For English speakers the NPS was “great” and it was considered “excellent” among Spanish speakers. Additionally, we observed high engagement with the HMP from both English and Spanish speakers. It is well-documented that non-English speaking populations are at risk for significant disparities in the delivery of telemedicine-based care [36–38]. This data demonstrates that telemedicine programs that are offered in multiple language and are designed with interpretive services as a centerpiece of their care can help overcome these disparities and achieve high engagement and satisfaction among non-English speakers.

A key aim of healthcare is keeping people connected to outpatient ambulatory care [39]. This connection is especially important as patients’ experience health challenges including COVID-19. Our results show that the COVID-19 positive patients in HMP had increased likelihood of having outpatient care within 7 and 30 days from their COVID-19 test compared to propensity weighted COVID-19 patients who received usual care. Use of primary care, a subset of outpatient care, was increased at 30 days. Again, this pattern of increased use of care was seen for English and Spanish-speaking patients. Disparities in access to care between Spanish and English speakers are well-documented [40, 41]. The similar increased use of care for Spanish speakers suggests that language-concordant care in telemedicine may help to improve access to care. Overall, the HMP COVID-19 patient increased use of care suggests that the HMP helped keep COVID-19 patients connected to outpatient care.

We also observed an increased use of ED care among COVID-19 HMP patients compared to propensity weighted COVID-19 positive patients who received usual care. This result is different than a recent study that showed decreased ED use for COVID-19 patients engaged in a remote monitoring program compared to those who declined to participate, but the study also suggests that this may have been due to differences in the groups at baseline such their current health status [11]. Our propensity models aimed to account for COVID-19 disease severity at presentation and comorbidities across groups, which suggests that the health status at the time of testing should be similar. Thus, increased ED use should not be due to COVID-19 positive HMP patients starting out sicker than COVID-19 patients who did not use the HMP. However, the health system is still learning how to predict which COVID-19 patients will have their condition deteriorate and we may not have been able to account for these factors. In addition, early treatment of COVID while the patient requires only low flow oxygen has been shown to improve mortality, so an increase in ED visits at the appropriate time may potentially improve clinical outcomes [42].We postulate that the increased monitoring of patient symptoms may have led to increased referrals to the ED. More research is needed to understand how home monitoring of COVID-19 patients impacts ED referral, appropriate thresholds for referrals, clinical outcomes, and patient reported outcomes.

This study has several key limitations. Our main data source for use of care was Providence electronic health records. Thus, we were not able to measure use of outpatient, primary, or ED care outside of the Providence health system. There is potential for sample bias based on type of patient more likely to engage in telemedicine. Our propensity weighting accounted for a multitude of characteristics to align our treatment and control groups, but we were still limited by information available in electronic health records and could not account for other factors such as social determinants of health. Finally, we did not collect any information on patient recovery and COVID-19 outcomes as this was beyond the scope of the study but should be the focus of future studies.

## Conclusion

Effective ways to monitor COVID-19 patients from home remains a top priority as health systems continue to meet the challenge of COVID-19 variants and shifting public health policies and practices. Our results show a high satisfaction for patients engaged in the Providence multi-lingual COVID-19 HMP and high level of comfort using at home monitoring devices. We show that the COVID-19 HMP increased use of outpatient care, which indicates an increased connection to care. We also observed an increased use of the ED. Finally, our results were similar across English and Spanish-speakers, suggesting that language-appropriate telemedicine interventions can help bridge care disparities during public health crises and facilitate equity in healthcare utilization. Additional study is required to better understand the impact of language- appropriate telemedicine more broadly.

## Supporting information

S1 Data (DOCX)

S2 Data (ZIP)
