# Supplementary material for: Patient experience and healthcare utilization for a COVID-19 telemedicine home monitoring program offered in English and Spanish
Source: PLoS One. 2022 Jun 30;17(6):e0270754. doi: 10.1371/journal.pone.0270754 (PMC9246185; doi:10.1371/journal.pone.0270754)
Supplement: S1 Data — (DOCX) [file pone.0270754.s001.docx]

Supplementary Data

**Table 1A. Characteristics of Cases and Controls before and after propensity weighting**

| **Characteristic** | **Full Study Population** | | **Propensity Population (Unweighted)** | | **Propensity Population (weighted)** | |
| --- | --- | --- | --- | --- | --- | --- |
|  | **Case - High Touch**, N = 4,358 | **Control**, N = 345,157 | **Case - High Touch**, N = 4,288 | **Control**, N = 165,316 | **Case - High Touch**, N = 4,288 | **Control**, N = 156,968 |
| **Age^1^** | 54 (17) | 51 (20) | 54 (17) | 49 (19) | 54 (17) | 53 (19) |
| **Age Group** |  |  |  |  |  |  |
| 18 to 34 | 691 (15.9%) | 92,781 (26.9%) | 690 (16.1%) | 48,180 (29.1%) | 690 (16.1%) | 30,855 (19.7%) |
| 35 to 44 | 672 (15.4%) | 52,229 (15.1%) | 668 (15.6%) | 26,976 (16.3%) | 668 (15.6%) | 24,753 (15.8%) |
| 45 to 54 | 843 (19.3%) | 48,131 (13.9%) | 839 (19.6%) | 25,401 (15.4%) | 839 (19.6%) | 28,044 (17.9%) |
| 55 to 64 | 878 (20.1%) | 54,288 (15.7%) | 866 (20.2%) | 25,896 (15.7%) | 866 (20.2%) | 28,767 (18.3%) |
| 65 to 74 | 737 (16.9%) | 49,891 (14.5%) | 715 (16.7%) | 20,848 (12.6%) | 715 (16.7%) | 22,532 (14.4%) |
| 75+ | 537 (12.3%) | 47,837 (13.9%) | 510 (11.9%) | 18,015 (10.9%) | 510 (11.9%) | 22,017 (14.0%) |
| **Sex** |  |  |  |  |  |  |
| Female | 2,254 (51.7%) | 197,351 (57.2%) | 2,229 (52.0%) | 92,176 (55.8%) | 2,229 (52.0%) | 83,823 (53.4%) |
| Male | 2,104 (48.3%) | 147,740 (42.8%) | 2,059 (48.0%) | 73,140 (44.2%) | 2,059 (48.0%) | 73,145 (46.6%) |
| Other | 0 (0.0%) | 49 (0.0%) |  |  |  |  |
| Unknown | 0 (0.0%) | 17 (0.0%) |  |  |  |  |
| **Ethnicity** |  |  |  |  |  |  |
| Hispanic or Latino | 1,467 (33.7%) | 43,193 (12.5%) | 1,450 (33.8%) | 25,443 (15.4%) | 1,450 (33.8%) | 49,939 (31.8%) |
| Non-Hispanic | 2,810 (64.5%) | 284,150 (82.3%) | 2,758 (64.3%) | 131,303 (79.4%) | 2,758 (64.3%) | 103,887 (66.2%) |
| Unknown | 81 (1.9%) | 17,814 (5.2%) | 80 (1.9%) | 8,570 (5.2%) | 80 (1.9%) | 3,142 (2.0%) |
| **Race** |  |  |  |  |  |  |
| American Indian or Alaska Native | 41 (0.9%) | 4,181 (1.2%) | 40 (0.9%) | 1,761 (1.1%) | 40 (0.9%) | 1,554 (1.0%) |
| Asian | 207 (4.8%) | 18,125 (5.3%) | 204 (4.8%) | 8,242 (5.0%) | 204 (4.8%) | 7,201 (4.6%) |
| Black | 214 (4.9%) | 14,376 (4.2%) | 213 (5.0%) | 7,883 (4.8%) | 213 (5.0%) | 7,925 (5.1%) |
| Hispanic or Latino | 1,467 (33.7%) | 43,193 (12.5%) | 1,450 (33.8%) | 25,443 (15.4%) | 1,450 (33.8%) | 49,939 (31.8%) |
| Native Hawaiian or Other Pacific Islander | 100 (2.3%) | 2,818 (0.8%) | 98 (2.3%) | 1,450 (0.9%) | 98 (2.3%) | 3,328 (2.1%) |
| Other | 213 (4.9%) | 12,703 (3.7%) | 209 (4.9%) | 6,659 (4.0%) | 209 (4.9%) | 7,576 (4.8%) |
| Unknown | 111 (2.5%) | 17,903 (5.2%) | 110 (2.6%) | 8,332 (5.1%) | 110 (2.6%) | 4,129 (2.6%) |
| White | 2,001 (46.0%) | 231,025 (67.1%) | 1,960 (45.8%) | 105,094 (63.7%) | 1,960 (45.8%) | 75,158 (47.9%) |
| Unknown | 4 | 833 | 4 | 452 | 4 | 159 |
| **Language** |  |  |  |  |  |  |
| English | 3,292 (75.5%) | 320,467 (92.8%) | 3,240 (75.6%) | 152,332 (92.1%) | 3,240 (75.6%) | 123,108 (78.4%) |
| Other | 269 (6.2%) | 9,769 (2.8%) | 263 (6.1%) | 4,555 (2.8%) | 263 (6.1%) | 8,704 (5.5%) |
| Spanish | 793 (18.2%) | 13,154 (3.8%) | 781 (18.2%) | 7,739 (4.7%) | 781 (18.2%) | 24,980 (15.9%) |
| Unknown | 4 (0.1%) | 1,767 (0.5%) | 4 (0.1%) | 690 (0.4%) | 4 (0.1%) | 176 (0.1%) |
| **Payor Type** |  |  |  |  |  |  |
| Capitation | 164 (3.8%) | 8,905 (2.6%) | 157 (3.7%) | 5,577 (3.4%) | 157 (3.7%) | 6,022 (3.8%) |
| Commercial | 1,309 (30.0%) | 140,940 (40.8%) | 1,293 (30.2%) | 68,912 (41.7%) | 1,293 (30.2%) | 47,382 (30.2%) |
| Managed Care | 122 (2.8%) | 7,515 (2.2%) | 121 (2.8%) | 3,437 (2.1%) | 121 (2.8%) | 4,695 (3.0%) |
| Medicaid | 1,108 (25.4%) | 59,993 (17.4%) | 1,103 (25.7%) | 31,212 (18.9%) | 1,103 (25.7%) | 40,397 (25.7%) |
| Medicare | 1,185 (27.2%) | 96,097 (27.8%) | 1,147 (26.7%) | 38,304 (23.2%) | 1,147 (26.7%) | 41,993 (26.8%) |
| Other | 355 (8.1%) | 14,063 (4.1%) | 353 (8.2%) | 7,642 (4.6%) | 353 (8.2%) | 12,071 (7.7%) |
| Self-pay | 115 (2.6%) | 17,644 (5.1%) | 114 (2.7%) | 10,232 (6.2%) | 114 (2.7%) | 4,408 (2.8%) |
| **Encounter Setting** |  |  |  |  |  |  |
| ED | 2,069 (47.5%) | 55,501 (16.1%) | 2,038 (47.5%) | 39,734 (24.0%) | 2,038 (47.5%) | 80,792 (51.5%) |
| IP | 1,384 (31.8%) | 96,776 (28.0%) | 1,360 (31.7%) | 26,002 (15.7%) | 1,360 (31.7%) | 37,699 (24.0%) |
| OP | 749 (17.2%) | 170,524 (49.4%) | 736 (17.2%) | 92,159 (55.7%) | 736 (17.2%) | 31,971 (20.4%) |
| Virtual | 156 (3.6%) | 22,356 (6.5%) | 154 (3.6%) | 7,421 (4.5%) | 154 (3.6%) | 6,506 (4.1%) |
| **Final COVID status** |  |  |  |  |  |  |
| Negative | 1,795 (41.2%) | 213,635 (61.9%) | 1,763 (41.1%) | 108,393 (65.6%) | 1,763 (41.1%) | 66,864 (42.6%) |
| Positive | 2,456 (56.4%) | 23,874 (6.9%) | 2,423 (56.5%) | 13,102 (7.9%) | 2,423 (56.5%) | 78,468 (50.0%) |
| PUI | 0 (0.0%) | 101 (0.0%) |  |  |  |  |
| Unknown | 107 (2.5%) | 107,547 (31.2%) | 102 (2.4%) | 43,821 (26.5%) | 102 (2.4%) | 11,636 (7.4%) |
| **Facility State** |  |  |  |  |  |  |
| Alaska | 159 (3.7%) | 13,008 (3.8%) | 158 (3.7%) | 3,680 (2.2%) | 158 (3.7%) | 6,129 (3.9%) |
| California | 1,238 (28.4%) | 79,081 (23.0%) | 1,219 (28.4%) | 36,864 (22.4%) | 1,219 (28.4%) | 47,132 (30.0%) |
| Idaho | 0 (0.0%) | 4 (0.0%) |  |  |  |  |
| Montana | 45 (1.0%) | 10,777 (3.1%) | 44 (1.0%) | 2,436 (1.5%) | 44 (1.0%) | 1,871 (1.2%) |
| Oregon | 538 (12.4%) | 70,030 (20.3%) | 531 (12.4%) | 24,666 (15.0%) | 531 (12.4%) | 20,443 (13.0%) |
| Washington | 2,376 (54.5%) | 171,491 (49.8%) | 2,334 (54.5%) | 97,185 (59.0%) | 2,334 (54.5%) | 81,315 (51.8%) |
| Unknown | 2 | 766 | 2 | 485 | 2 | 77 |
| **Department Specialty Group at Dx** |  |  |  |  |  |  |
| ED | 2,071 (47.5%) | 55,959 (16.2%) | 2,039 (47.6%) | 40,045 (24.2%) | 2,039 (47.6%) | 81,050 (51.7%) |
| Other | 1,224 (28.1%) | 117,855 (34.1%) | 1,201 (28.0%) | 26,090 (15.8%) | 1,201 (28.0%) | 37,681 (24.0%) |
| Primary Care | 473 (10.9%) | 50,543 (14.6%) | 469 (10.9%) | 19,166 (11.6%) | 469 (10.9%) | 13,749 (8.8%) |
| Urgent care | 588 (13.5%) | 120,777 (35.0%) | 577 (13.5%) | 80,004 (48.4%) | 577 (13.5%) | 24,420 (15.6%) |
| Unknown | 2 | 23 | 2 | 11 | 2 | 67 |
| **Marital Status** |  |  |  |  |  |  |
| Divorced | 332 (7.6%) | 23,241 (6.7%) | 331 (7.7%) | 10,817 (6.5%) | 331 (7.7%) | 12,068 (7.7%) |
| Domestic Partner | 5 (0.1%) | 648 (0.2%) | 5 (0.1%) | 280 (0.2%) | 5 (0.1%) | 195 (0.1%) |
| Legally Separated | 73 (1.7%) | 3,324 (1.0%) | 73 (1.7%) | 1,709 (1.0%) | 73 (1.7%) | 2,597 (1.7%) |
| Married | 2,223 (51.0%) | 155,330 (45.0%) | 2,182 (50.9%) | 70,317 (42.5%) | 2,182 (50.9%) | 76,955 (49.0%) |
| Patient Refused | 0 (0.0%) | 85 (0.0%) |  |  |  |  |
| Significant Other | 61 (1.4%) | 5,588 (1.6%) | 60 (1.4%) | 2,636 (1.6%) | 60 (1.4%) | 2,340 (1.5%) |
| Single | 1,318 (30.2%) | 122,215 (35.4%) | 1,311 (30.6%) | 64,213 (38.8%) | 1,311 (30.6%) | 50,708 (32.3%) |
| Unknown | 90 (2.1%) | 13,542 (3.9%) | 88 (2.1%) | 6,920 (4.2%) | 88 (2.1%) | 3,529 (2.2%) |
| Widowed | 256 (5.9%) | 21,184 (6.1%) | 238 (5.6%) | 8,424 (5.1%) | 238 (5.6%) | 8,577 (5.5%) |
| **Smoking Status** |  |  |  |  |  |  |
| Current | 433 (9.9%) | 44,378 (12.9%) | 422 (9.8%) | 22,609 (13.7%) | 422 (9.8%) | 17,336 (11.0%) |
| Former | 1,100 (25.2%) | 86,082 (24.9%) | 1,071 (25.0%) | 38,166 (23.1%) | 1,071 (25.0%) | 39,164 (25.0%) |
| Never Smoker | 2,638 (60.5%) | 193,973 (56.2%) | 2,610 (60.9%) | 92,041 (55.7%) | 2,610 (60.9%) | 92,812 (59.1%) |
| Unknown | 187 (4.3%) | 20,724 (6.0%) | 185 (4.3%) | 12,500 (7.6%) | 185 (4.3%) | 7,656 (4.9%) |
| **Past Medical History** |  |  |  |  |  |  |
| Chronic Obstructive Pulmonary Disease | 64 (1.5%) | 5,841 (1.7%) | 59 (1.4%) | 2,033 (1.2%) | 59 (1.4%) | 2,349 (1.5%) |
| Diabetes Mellitus | 378 (8.7%) | 15,937 (4.6%) | 374 (8.7%) | 5,449 (3.3%) | 374 (8.7%) | 9,887 (6.3%) |
| Hypertension | 483 (11.1%) | 27,885 (8.1%) | 473 (11.0%) | 9,074 (5.5%) | 473 (11.0%) | 13,257 (8.4%) |
| Coronary Artery Disease | 114 (2.6%) | 10,423 (3.0%) | 108 (2.5%) | 3,322 (2.0%) | 108 (2.5%) | 3,586 (2.3%) |
| Hepatitis B | 1 (0.0%) | 113 (0.0%) | 1 (0.0%) | 30 (0.0%) | 1 (0.0%) | 27 (0.0%) |
| Cancer | 49 (1.1%) | 7,314 (2.1%) | 45 (1.0%) | 1,743 (1.1%) | 45 (1.0%) | 1,613 (1.0%) |
| Chronic Kidney Disease | 153 (3.5%) | 9,421 (2.7%) | 148 (3.5%) | 3,225 (2.0%) | 148 (3.5%) | 4,463 (2.8%) |
| Immunodeficiency | 12 (0.3%) | 380 (0.1%) | 12 (0.3%) | 112 (0.1%) | 12 (0.3%) | 340 (0.2%) |
| Asthma | 69 (1.6%) | 3,310 (1.0%) | 69 (1.6%) | 1,032 (0.6%) | 69 (1.6%) | 1,801 (1.1%) |
| Congestive Heart Failure | 94 (2.2%) | 10,167 (2.9%) | 90 (2.1%) | 3,419 (2.1%) | 90 (2.1%) | 3,289 (2.1%) |
| Obstructive Sleep Apnea | 67 (1.5%) | 3,580 (1.0%) | 65 (1.5%) | 1,074 (0.6%) | 65 (1.5%) | 1,827 (1.2%) |
| Rheumatoid Arthritis | 22 (0.5%) | 1,756 (0.5%) | 21 (0.5%) | 547 (0.3%) | 21 (0.5%) | 809 (0.5%) |
| End State Renal Disease | 43 (1.0%) | 1,822 (0.5%) | 41 (1.0%) | 686 (0.4%) | 41 (1.0%) | 1,185 (0.8%) |
| Cirrhosis | 12 (0.3%) | 1,645 (0.5%) | 11 (0.3%) | 493 (0.3%) | 11 (0.3%) | 400 (0.3%) |
| Transplant history | 17 (0.4%) | 433 (0.1%) | 17 (0.4%) | 104 (0.1%) | 17 (0.4%) | 360 (0.2%) |
| Coagulopathy | 14 (0.3%) | 1,190 (0.3%) | 13 (0.3%) | 345 (0.2%) | 13 (0.3%) | 369 (0.2%) |
| **Reason for Visit (presenting symptoms)** |  |  |  |  |  |  |
| Chills | 126 (2.9%) | 4,745 (1.4%) | 125 (2.9%) | 2,939 (1.8%) | 125 (2.9%) | 4,862 (3.1%) |
| Congestion | 71 (1.6%) | 8,992 (2.6%) | 71 (1.7%) | 5,330 (3.2%) | 71 (1.7%) | 2,804 (1.8%) |
| Cough | 1,505 (34.5%) | 35,525 (10.3%) | 1,483 (34.6%) | 21,228 (12.8%) | 1,483 (34.6%) | 50,374 (32.1%) |
| Fever | 1,133 (26.0%) | 21,979 (6.4%) | 1,115 (26.0%) | 12,335 (7.5%) | 1,115 (26.0%) | 37,067 (23.6%) |
| Flu like symptoms | 97 (2.2%) | 3,705 (1.1%) | 97 (2.3%) | 2,182 (1.3%) | 97 (2.3%) | 3,737 (2.4%) |
| GI symptoms | 329 (7.5%) | 19,473 (5.6%) | 323 (7.5%) | 10,750 (6.5%) | 323 (7.5%) | 12,048 (7.7%) |
| Headache | 206 (4.7%) | 12,922 (3.7%) | 206 (4.8%) | 7,931 (4.8%) | 206 (4.8%) | 8,015 (5.1%) |
| Hemoptysis | 13 (0.3%) | 356 (0.1%) | 13 (0.3%) | 194 (0.1%) | 13 (0.3%) | 404 (0.3%) |
| Infection | 68 (1.6%) | 3,709 (1.1%) | 68 (1.6%) | 2,688 (1.6%) | 68 (1.6%) | 2,924 (1.9%) |
| Myalgia | 261 (6.0%) | 8,869 (2.6%) | 254 (5.9%) | 5,559 (3.4%) | 254 (5.9%) | 9,582 (6.1%) |
| Reactive airway | 4 (0.1%) | 332 (0.1%) | 4 (0.1%) | 176 (0.1%) | 4 (0.1%) | 180 (0.1%) |
| Shortness of breath | 1,666 (38.2%) | 34,709 (10.1%) | 1,637 (38.2%) | 19,256 (11.6%) | 1,637 (38.2%) | 53,874 (34.3%) |
| Throat conditions | 169 (3.9%) | 19,694 (5.7%) | 167 (3.9%) | 12,124 (7.3%) | 167 (3.9%) | 6,799 (4.3%) |
| Wheezing | 8 (0.2%) | 562 (0.2%) | 8 (0.2%) | 335 (0.2%) | 8 (0.2%) | 330 (0.2%) |
| **Diagnosis (at presentation)** |  |  |  |  |  |  |
| Influenza | 22 (0.5%) | 954 (0.3%) | 22 (0.5%) | 421 (0.3%) | 22 (0.5%) | 928 (0.6%) |
| Infection that does not include respiratory distress | 637 (14.6%) | 33,442 (9.7%) | 626 (14.6%) | 15,229 (9.2%) | 626 (14.6%) | 22,125 (14.1%) |
| Pneumonia | 1,222 (28.0%) | 11,766 (3.4%) | 1,208 (28.2%) | 4,940 (3.0%) | 1,208 (28.2%) | 31,098 (19.8%) |
| Respiratory infection that is not pneumonia | 548 (12.6%) | 38,986 (11.3%) | 545 (12.7%) | 22,402 (13.6%) | 545 (12.7%) | 21,409 (13.6%) |
| COPD/ASTHMA | 165 (3.8%) | 6,512 (1.9%) | 157 (3.7%) | 3,431 (2.1%) | 157 (3.7%) | 5,988 (3.8%) |
| White Blood Cell Count | 57 (1.3%) | 3,875 (1.1%) | 57 (1.3%) | 1,553 (0.9%) | 57 (1.3%) | 1,906 (1.2%) |
| Vomiting, Diarrhea, Nausea | 238 (5.5%) | 16,992 (4.9%) | 236 (5.5%) | 9,094 (5.5%) | 236 (5.5%) | 8,605 (5.5%) |
| Cough | 705 (16.2%) | 30,898 (9.0%) | 697 (16.3%) | 17,341 (10.5%) | 697 (16.3%) | 26,352 (16.8%) |
| Shortness of Breath | 1,329 (30.5%) | 31,230 (9.0%) | 1,307 (30.5%) | 14,320 (8.7%) | 1,307 (30.5%) | 38,383 (24.5%) |
| Wheezing | 26 (0.6%) | 1,045 (0.3%) | 26 (0.6%) | 590 (0.4%) | 26 (0.6%) | 1,041 (0.7%) |
| Pain in Throat | 3 (0.1%) | 312 (0.1%) | 3 (0.1%) | 215 (0.1%) | 3 (0.1%) | 123 (0.1%) |
| Nasal Congestion | 15 (0.3%) | 4,287 (1.2%) | 15 (0.3%) | 2,111 (1.3%) | 15 (0.3%) | 644 (0.4%) |
| Fever | 555 (12.7%) | 16,996 (4.9%) | 547 (12.8%) | 9,005 (5.4%) | 547 (12.8%) | 18,587 (11.8%) |
| Enlarged Lymph Nodes | 2 (0.0%) | 598 (0.2%) | 2 (0.0%) | 235 (0.1%) | 2 (0.0%) | 88 (0.1%) |
| Chills w/o Fever | 34 (0.8%) | 2,109 (0.6%) | 34 (0.8%) | 1,196 (0.7%) | 34 (0.8%) | 1,407 (0.9%) |
| **PSI Score** | 63 (32) | 58 (36) | 62 (31) | 52 (33) | 62 (31) | 60 (34) |
| **Utilization History (Last 12 Months)** |  |  |  |  |  |  |
| ED Visits | 1,247 (28.6%) | 56,100 (16.3%) | 1,225 (28.6%) | 29,681 (18.0%) | 1,225 (28.6%) | 43,622 (27.8%) |
| Inpatient Admissions | 513 (11.8%) | 32,819 (9.5%) | 487 (11.4%) | 13,158 (8.0%) | 487 (11.4%) | 17,925 (11.4%) |
| Outpatient Visits | 1,999 (45.9%) | 131,435 (38.1%) | 1,956 (45.6%) | 64,439 (39.0%) | 1,956 (45.6%) | 65,678 (41.8%) |
| Virtual Visits | 341 (7.8%) | 27,189 (7.9%) | 334 (7.8%) | 13,416 (8.1%) | 334 (7.8%) | 10,276 (6.5%) |
| PCP Visits | 1,149 (26.4%) | 77,973 (22.6%) | 1,123 (26.2%) | 40,736 (24.6%) | 1,123 (26.2%) | 37,467 (23.9%) |
